# Book Reviews

**Published:** 1903-06

**Authors:** 


### BOOK REVIEWS.

DISEASES OF THE HEART AND ARTERIAL SYSTEM: Being a Practical Presentation of the Subject, for the Use of Students and Practitioners of Medicine. By Robert H. Babcock, A.M., M.D., Chicago; with three colored plates and one hundred and ninety-nine illustrations. D. Appleton & Co. New York and London.

This elaborate work is divided into sections dealing with general considerations of the anatomy, physiology and examination of the heart, diseases of the pericardium, diseases of the endocardium, diseases of the myocardium, cardiac neuroses, diseases of the arterial system, and an appendix concerned with mechanical devices for cardiac diagnostic work, the X-ray, the sphygmograph and Gaertner's tonometer. The book is admirably illustrated and the text clear and practical. Considering the physical disability—that of blindness—under which the author labors, or rather to which he rises superior, the book is a marvel. Apart from this, on its own merit, none better has appeared in English.

A PRACTICAL TREATISE ON MATERIA MEDICA AND THERAPEUTICS. By Roberts Bartholow, M.A., M.D., LL.D. Eleventh edition, revised and enlarged. D. Appleton & Co. New York and London, 1903.

Bartholow's Materia Medica needs no commendation at our hands. It has stood the test of time and its popularity is attested by the issuance of an eleventh edition.

The same general plan of the former editions is retained in the present one, with such emendations and additions as the progress of medicine has necessitated.

To those who are yet unfamiliar with this book we can commend it in no higher term than that of being an American classic.

